# Diagnostic Accuracy in Acute Venous Thromboembolism: Comparing D-Dimer, Thrombin Generation, Overall Hemostatic Potential, and Fibrin Monomers

**DOI:** 10.1055/s-0040-1714210

**Published:** 2020-08-20

**Authors:** Maria Farm, Aleksandra Antovic, David E. Schmidt, Niklas Bark, Nida Soutari, Anwar J. Siddiqui, Margareta Holmström, Iva Pruner, Jovan P. Antovic

**Affiliations:** 1 Department of Molecular Medicine and Surgery, Karolinska Institutet, Stockholm, Sweden; 2 Clinical Chemistry, Karolinska University Hospital, Stockholm, Sweden; 3 Rheumatology Unit, Department of Medicine, Karolinska Institutet, Stockholm, Sweden; 4 Academic Specialist Center, Center for Rheumatology, Stockholm Health Services, Stockholm, Sweden; 5 Department of Medicine, Solna, Karolinska Institutet, Stockholm, Sweden; 6 Coagulation Unit, Division of Haematology, Karolinska University Hospital, Stockholm, Sweden; 7 Emergency Medicine Function, Karolinska University Hospital, Stockholm, Sweden; 8 Division of Diagnostics and Specialist Medicine, Unit of Internal Medicine, Medicine and Caring Sciences, Department of Health, Linköping University, Linköping, Sweden

**Keywords:** predictive value of tests, global hemostatic assays, clinical studies, deep vein thromboses, pulmonary embolism

## Abstract

**Introduction** For acute venous thromboembolism (VTE), a biomarker with higher specificity than D-dimer would be of great clinical use. Thrombin generation and overall hemostatic potential (OHP) reflect the hemostatic balance by globally assessing multiple coagulation factors and inhibitors. These tests discriminate between healthy controls and patients with a prothrombotic tendency but have yet to be established as clinical biomarkers of VTE.

**Objective** This study compares endogenous thrombin potential (ETP) and OHP to D-dimer and fibrin monomers (FM) in outpatients with suspected VTE.

**Methods** A cross-sectional diagnostic study where 954 patients with suspected pulmonary embolism or deep venous thrombosis were recruited consecutively from the medical emergency department at Karolinska University Hospital. D-dimer, FM, OHP, and ETP were analyzed in a subpopulation of 60 patients with VTE and 98 matched controls without VTE. VTE was verified either by ultrasonography or computed tomography and clinical data were collected from medical records.

**Results** Compared with healthy controls, both VTE and non-VTE patients displayed prothrombotic profiles in OHP and ETP. D-dimer, FM, ETP area under the curve (AUC), and ETP T _lag_ were significantly different between patients with VTE and non-VTE. The largest receiver-operating characteristic AUCs for discrimination between VTE and non-VTE, were found in D-dimer with 0.94, FM 0.77, and ETP AUC 0.65. No useful cutoff could be identified for the ETP or the OHP assay.

**Conclusion** Compared with D-dimer, neither ETP nor OHP were clinically viable biomarkers of acute venous thrombosis. The data indicated that a large portion of the emergency patients with suspected VTE were in a prothrombotic state.

## Introduction

Venous thromboembolism (VTE) is the third most common cardiovascular disease and a cause of substantial morbidity and mortality worldwide. 1 A definite diagnosis of VTE can generally not be reached clinically, so the quality of assays and imaging techniques are of utmost importance. While imaging is needed to verify VTE, the use of biomarkers in select cases can reduce time, cost, and iatrogenic complications in the diagnostic process. 2 3 The only biomarker in common clinical use for diagnosis of VTE is D-dimer, the degradation product of polymerized and cross-linked fibrin. Fibrin monomers (FM) can possibly improve diagnosis if used together with D-dimer for the exclusion of VTE. 4 The specificity of D-dimer is low at the chosen cutoff, with false positive results in up to 30% of tested patients with suspected VTE. 5 The low specificity is partially due to increased levels in patients with comorbidities. 6 Increasing the effectiveness of the diagnostic process could potentially have major clinical and economical beneficial effects on the management of VTE.

Global hemostatic assays (GHAs) are a class of assays that examine the combined effect of pro- and anticoagulant processes in patient samples ex vivo. These assays monitor the whole coagulation process, either by using the clotting of whole blood or the generation of thrombin or fibrin in plasma as endpoints. While D-dimer reflects the in vivo activation of both hemostasis and fibrinolysis, the GHA illustrate the patients' current potential for these mechanisms. There is also the addition of a kinetic element, because the GHA continuously measure thrombin or fibrin generation and even fibrinolysis. Compared with D-dimer, global thrombin generation assays (TGAs) are also less influenced by comorbidities such as cancer, infectious disease, and cardiovascular disease. 7 8 A few studies have evaluated the use of TGA as a complement to D-dimer in the exclusion of VTE 9 10 and indicated promising results of increased specificity paired with maintained sensitivity. TGA have showed prolonged time to peak (T _max_ ) and time to initiation of coagulation (T _lag_ ) in acute VTE, possibly related to consumption of coagulation factors in the formation of the thrombus. 8 9 10 11 12 In contrast, other studies have found an increased total thrombin generating capacity measured as the area under the curve (AUC), 8 9 which would not be the case after consumption of coagulation factors. Similarly, an increased thrombin generating capacity has been demonstrated in intermediate and serious thrombophilic phenotypes 13 and has been associated with an increased risk of first VTE and unprovoked recurrent VTE, 14 15 although there are conflicting results. 16

In contrast to TGA, assays measuring the generation of fibrin, such as in the overall hemostatic potential (OHP) method, will also reflect patients' fibrinogen levels. 17 The OHP can potentially be used for screening both hypo- and hypercoagulable conditions. 18 19 It can also be used to characterize hypercoagulability or hypofibrinolysis in several prothrombotic conditions, such as antiphospholipid syndrome, 20 after VTE, 21 22 in acute coronary syndrome, 23 and in acute stroke. 24 Only a few studies have studied global fibrin generation in (semi-)acute venous thrombosis, these have demonstrated hypercoagulability and hypofibrinolysis compared with healthy controls. 25 26

The Innovance endogenous thrombin potential (ETP) 27 and the OHP 28 assays are GHAs that have been optimized for analysis in routine coagulation laboratories. The OHP is a manual method that has been modified for routine laboratories and the ETP is an automated chromogenic TGA. Both assays feature simplified preanalytical handling and shortened analysis time, rendering them potentially useful in acute settings.

There is significant room for improvement of the biochemical diagnosis of VTE. Although some data point to the potential usefulness of the GHAs, a lack of standardization has hampered progress 29 and the clinical diagnostic value of ETP and OHP for diagnosis of VTE has not been formally assessed in a real-world clinical setting. This cross-sectional diagnostic study compares the diagnostic accuracy of OHP and ETP to D-dimer and FM for the assessment of suspect acute VTE in outpatients contacting the emergency department.

## Materials and Methods

### Patients

Samples were taken from the Karolinska Age Adjusted D-Dimer study (DFW-VTE). 5 In short, 954 consecutive outpatients with clinically suspected pulmonary embolism (PE) or deep venous thrombosis (DVT) in the lower limb were prospectively recruited from the emergency department of Karolinska University Hospital in Huddinge, Sweden, between April 2014 and May 2015. Medical students were separately enrolled as healthy controls for post hoc analysis. The study was conducted in accordance with the Declaration of Helsinki and approved by the Regional Ethics Review Board in Stockholm (DNR 2013–2143–31–2). All participants provided written informed consent at enrolment.

### Samples

Plasma samples were collected in 0.109 mol/L (3.2%) sodium-citrate plastic vacutainer tubes (Becton Dickinson, New Jersey, United States) by direct venipuncture, without addition of corn trypsin inhibitor. 29 Samples were collected at the emergency unit before initiation of any anticoagulant therapy. After centrifugation at 3,000 × *g* for 10 minutes, samples were analyzed for D-dimer, FM, and prothrombin time (PT) (international normalized ratio [INR]) and then aliquoted and frozen at –70°C within 1 hour. Samples were thawed in a 37°C water bath for analysis of ETP, OHP, fibrinogen, antithrombin, and C-reactive protein (CRP). Because sample collection was performed in a routine clinical chemistry laboratory where storage of research samples could not be the main priority, only a subset of samples from the DFW-VTE study 5 could be saved for analysis by FM and stored.

**Fig. 1:**
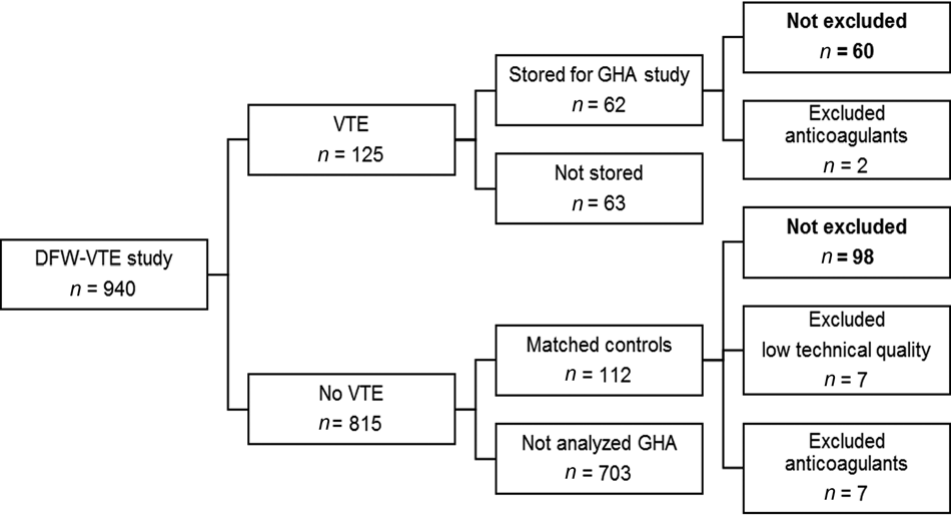
Flowchart of study samples. Matched controls were selected for all patients with venous thromboembolism (VTE). Only a subset of the 940 samples could be stored in the acute clinical chemistry laboratory. All included samples ( *n* = 174) were analyzed by overall hemostatic potential (OHP) and endogenous thrombin potential (ETP). Nine patients were excluded due to prior anticoagulant treatment and seven due to low technical quality. Primary analysis in this study was performed on 60 samples with VTE and 98 without VTE.

In this study, we included all samples with VTE and randomly selected age- and sex-matched samples without VTE ( Fig. 1 ). All included samples that were available (that had been stored) were analyzed by ETP and OHP ( *n* = 174; 62 VTE and 112 without VTE). After exclusions 158 samples remained, as follows ( Fig. 1 ). Seven non-VTE samples were excluded because of low technical quality due to hemolysis, lipemia, or clotting. Nine patients were excluded due to anticoagulant treatment, which was initiated before arrival to the emergency department. In four samples, ETP was not analyzed due to insufficient sample volume (one VTE, three non-VTE).

Healthy controls were also analyzed for OHP and fibrinogen ( *n* = 42) and ETP ( *n* = 37) to further characterize the hypercoagulable state of the study subjects.

### Clinical Data

Clinical data were collected from medical records by a resident MD blinded for the assay results. VTE had been verified radiologically by computed tomography or ultrasonography, as appropriate. VTE had been excluded radiologically ( *n* = 49) and was otherwise excluded by a 3-month follow-up of medical records ( *n* = 49). Radiology was accredited according to ISO/IEC 17025 by the Swedish Board for Accreditation and Conformity Assessment and was performed on the day of sampling in 83 patients, the following day in 15 patients, 3 days after sampling in 3 patients and after 7 days in 2 patients. Isolated thrombophlebitis was classified as negative for VTE and was present in nine cases. Patient characteristics are described in Table 1 . The eight patients with cancer suffered from prostate cancer ( *n* = 2), brain tumors ( *n* = 2), malignant melanoma ( *n* = 1), ovarian cancer ( *n* = 1), hairy cell leukemia ( *n* = 1), and liver metastasized cancer of unknown primary tumor ( *n* = 1). Recent trauma or surgery ( *n* = 7) was defined as an incident occurring less than 1 month before sampling. Prior thrombophilia testing in tested patients ( *n* = 17), had consisted of lupus anticoagulant, antithrombin, protein S, protein C, and the genetic variants Factor V Leiden and Factor II G20210A.

**Table 1:** Patient characteristics for patients with and without VTE

		No VTE ( *n* = 98)	VTE ( *n* = 60)	*p* -Value
Age [y]	Mean (95% CI), range	61 (57–64) 24–91	62 (58–66) 20–96	0.668 a
PT (INR) [< 1.2]	Median (IQR), range ( *n* )	1.0 (1.00–1.10) 0.8–1.5 (89)	1.0 (1.0–1.10) 0.98–1.10 (57)	0.419 b
Antithrombin, [0.8–1.2 kIU/L]	Median (IQR), range ( *n* )	1.0 (0.9–1.1) 0.6–1.5 (82)	1.0 (0.9–1.1) 0.6–1.4 (46)	0.194 b
Fibrinogen [2.0–4.2 g/L]	Median (IQR), range ( *n* )	3.5 (3.0–4.2) 1.0–6.0 (84)	4.0 (3.1–5.3) 1.2–7.5 (46)	0.057 b
CRP [< 3 mg/L]	Median (IQR), range ( *n* )	4 (1–11) 1–104 (98)	11 (4–44) 1–295 (59)	**< 0.001** b
Female	Proportion ( *n* )	0.48 (47)	0.42 (25)	0.441 c
Male	Proportion ( *n* )	0.52 (51)	0.58 (35)	
Previous VTE	Proportion ( *n* )	0.11 (11)	0.38 (23)	**< 0.001** c
Positive D-dimer	Proportion ( *n* )	0.34 (33)	0.97 (58)	**< 0.001** c
Trauma or surgery	Proportion ( *n* )	0.01 (1)	0.10 (6)	**0.012** d
Cancer	Proportion ( *n* )	0.08 (8)	0.07 (4)	1.000 d
Liver disease	Proportion ( *n* )	0.07 (7)	0.00 (0)	**0.045** d
Pregnant	Proportion ( *n* )	0.03 (3)	0.02 (1)	1.000 d
Platelet inhibitors	Proportion ( *n* )	0.39 (38)	0.22 (13)	**0.026** c
PHC	Proportion ( *n* )	0.02 (2)	0.05 (3)	0.369 d
Thrombophilia	Counts	2 of 10 tested	15 of 30 tested	

Abbreviations: CI, confidence interval; CRP, C-reactive protein; INR, international normalized ratio; IQR, interquartile range; PHC, prothrombotic hormonal contraceptives; PT, prothrombin time; VTE, venous thromboembolism.

Notes: No significance test was performed on the thrombophilia variable, because the patients without VTE had not been tested to a comparable extent.

Boldface values signify *p* -values for significant differences.

a Individual variables *t* -test.

b Mann–Whitney *U* test.

c Pearson's chi-square test.

d Fischer's exact test.

### Assays

Assays were performed blinded to clinical data and results of any other assay. Thrombin generation was analyzed using the automated Innovance ETP assay 27 30 on the BCS XP System. The instrument, reagent, and calibrator were provided by Siemens Healthcare Diagnostics (Erlangen, Germany). The ETP was performed in platelet-poor plasma using B-settings, the proprietary recommended settings for patients with suspected hypercoagulability, activated by tissue factor (TF) in high concentration (300 pm ). The reagent contains a nondefined fibrin aggregation inhibitor and a slow reacting thrombin chromophore substrate (H-β-Ala-Gly-Arg-pNA). The parameters are the area under the thrombin generation curve (ETP AUC), which corresponds to the total generation of thrombin, the peak thrombin concentration (ETP C _max_ ), the time to initiation of thrombin generation (ETP T _lag_ ), and the time to peak thrombin generation (ETP T _max_ ). Results were normalized against pooled normal plasma, giving results in %. Intra-assay and interassay variation coefficient (CV%) for the ETP AUC were 3.3 and 2.7%, respectively.

OHP was performed in 96-well plates using the method modified for routine laboratories, as previously described. 31 Two curves (with and without fibrinolysis initiated by tissue plasminogen activator) were used to calculate the area under the fibrin generation curve (overall coagulation potential, OCP), the AUC in the well of fibrin generation plus fibrinolysis (OHP), and the decrease in fibrinogen concentration by fibrinolysis as a proportion of the OCP (overall fibrinolytic potential, OFP). Intra-assay and interassay CV% for the OHP were 9.3 and 12.3%, respectively.

D-dimer, FM, and PT (INR) were analyzed immediately on the Sysmex CS2100i instrument (Siemens). D-dimer was analyzed using the Tina-quant D-dimer (Roche Diagnostics, Basel, Switzerland). 32 FM was analyzed using STA-Liatest FM (Diagnostica Stago, Asnières-sur-Seine, France). 33 Both assays are rapid particle-enhanced immunoturbidimetric assays. PT (INR) was analyzed by MRX Owrens PT (Medirox, Nyköping, Sweden). 34

Fibrinogen and antithrombin were analyzed on the Sysmex CS5100 instrument (Siemens) in all samples with remaining plasma after analysis of ETP and OHP. Fibrinogen was analyzed using the Dade thrombin reagent (Siemens) which is a modified Clauss assay. 35 Antithrombin was analyzed with enzymatic the FII-based Berichrom Antithrombin III (Siemens) assay. 36 CRP was analyzed with the immunoturbidimetric CRPL3, C-Reactive Protein Gen. 3 assay on the Cobas 6000 instrument (Roche Diagnostics). The exact number of samples that were available for each assay is given in Tables 1 and 2 .

**Table 2:** Assay results and significant differences between patients with and without VTE

Parameter	No VTE	VTE	*p* -Value	Reference interval c	Healthy controls, central 95 percentile
OHP, mean (±SEM)	15.9 (15.3–16.5)	16.2 (15.3–17.1)	0.811 a	6.5–13.6	6.3–16.2
OCP, mean (±SEM)	25.0 (24.3–25.7)	26.5 (38.5–41.3)	0.166 a	12.7–24.0	14.5–27.8
OFP, mean (±SEM)	37 (36–39)	40 (38–41)	0.166 a	31–57	38–58
ETP AUC, mean (±SEM)	99 (97–100)	107 (106–109)	** 0.001 a**	87–128	74–112
ETP C _max,_ median (IQR)	111 (99–122)	113 (103–127)	0.136 b	82–119	78–109
ETP t _lag_ , median (IQR)	21.2 (19.6–22.6)	22.0 (20.3–23.9)	** 0.031 b**	–	22–30
ETP t _max_ , mean (±SEM)	56.4 (55.6–57.2)	54.4 (53.5–55.4)	0.126 a	–	59–85
D-dimer, median (IQR)	0.38 (0.22–0.69)	2.93 (1.55–12.27)	** < 0.001 b**	< 0.5	–
Fibrin monomers, median (IQR)	3 (1–4)	9 (3–117)	** < 0.001 b**	< 6	–

Abbreviations: AUC, area under the curve; ETP, endogenous thrombin potential; IQR, interquartile range; OHP, overall hemostatic potential; SEM, standard error of the mean; VTE, venous thromboembolism.

Notes: With VTE, *n* = 98 (D-dimer, OHP, OCP, OFP) and *n* = 95 (fibrin monomers, ETP AUC, ETP C _max_ , ETP t _lag_ , ETP t _max_ ). Without VTE, *n* = 60 (D-dimer, OHP, OCP, OFP) and *n* = 59 (fibrin monomers, ETP AUC, ETP C _max_ , ETP t _lag_ , ETP t _max_ ).

Boldface values signify *p* -values for significant differences.

a Independent samples *t* -test.

b Mann–Whitney *U* test.

c Reference intervals OHP 31 and ETP. 27

### Statistical Methods

Descriptive statistics are presented as a range, mean (with 95% confidence interval [CI] or ± standard error of the mean), median, and interquartile range, as appropriate. Groups were compared using the independent samples *t* -test or by the nonparametric Mann–Whitney *U* test. Multiple groups were compared by a Kruskal–Wallis rank-sum test. Proportions were tested by Pearson's chi-square or Fischer's exact test. Pretest probabilities were calculated to assure diagnostic comparability between the assays. To summarize the overall discriminatory value of the assays, receiver-operating characteristic (ROC) AUCs (ROC AUCs) 37 were used. The diagnostic accuracy of the parameters of the OHP and ETP assays and FM were compared by sensitivity and specificity at the level where the sensitivity was equal to the sensitivity of D-dimer in the cohort. The association of ETP AUC with VTE status was examined by binomial logistic regression. ETP and OHP results were visually compared in clinical subgroups. Odds ratio (OR) for VTE was calculated for OCP, before and after adjusting for fibrinogen levels. Statistical analysis was performed using SPSS 23, MS Excel, and R version 3.6.0. *p* -Values < 0.05 were considered significant.

## Results

### Patient Characteristics

The study cohort included 60 patients with VTE and 98 randomly selected patients with non-VTE ( Fig. 1 ). VTE was localized as PE in 16 patients, proximal DVT in 25, and distal DVT, isolated below popliteal level in 19 patients. Thrombophlebitis was found in 10 patients and was classified as negative for VTE. Patient characteristics are summarized in Table 1 . Patients with VTE had significantly higher CRP results (median 11 vs. 4 mg/L, *p* < 0.001), frequency of positive D-dimer (0.97 vs. 0.34, *p* < 0.001), and prevalence of previous VTE (0.38 vs. 0.11, *p* < 0.001) and of recent trauma or surgery (0.10 vs. 0.01, *p* = 0.012). They also had a lower prevalence of treatment with platelet inhibitors (0.22 vs. 0.39, *p* = 0.026) and of liver disease (0.00 vs. 0.07, *p* = 0.045).

### Assessment of ETP and OHP in Patients with Suspected VTE

**Fig. 2:**
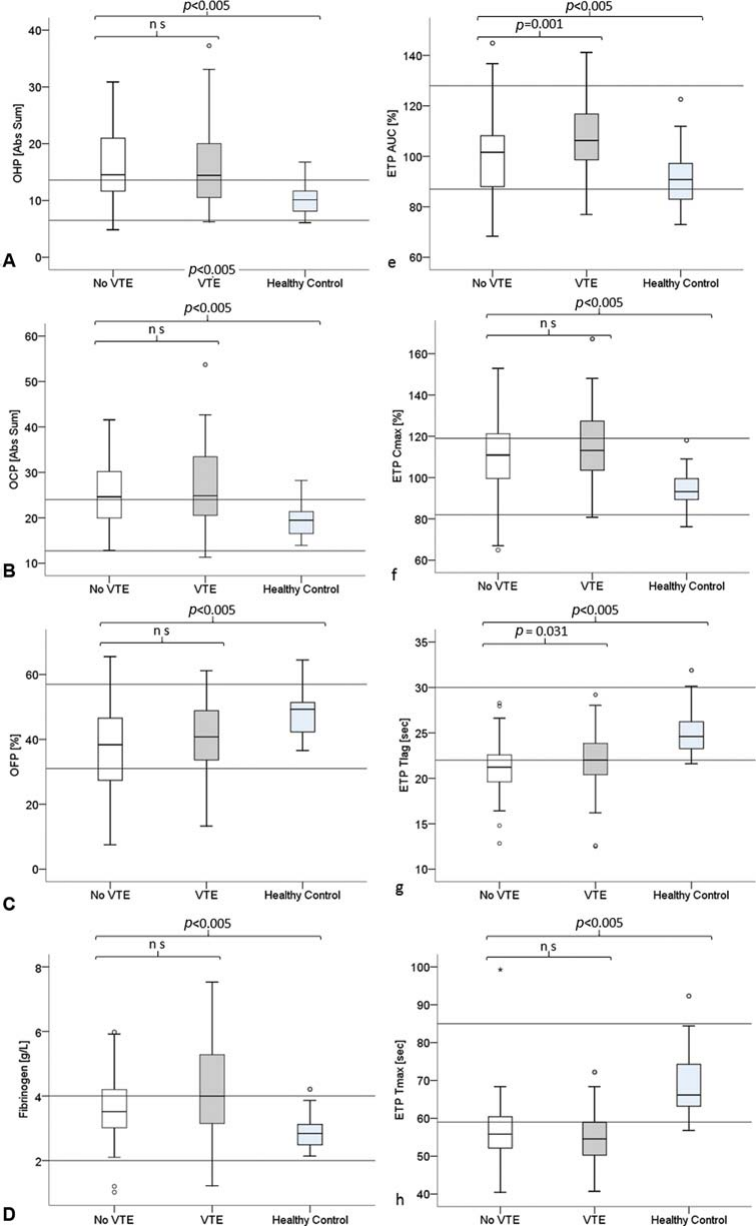
(A) Overall hemostatic potential (OHP), (B) OCP, (C) OFP, (D) fibrinogen, (E) endogenous thrombin potential area under the curve (ETP AUC), (F) ETP C _max_ , (G) ETP T _lag_ , and (H) ETP T _max_ . Difference in global hemostatic parameters between patients with no venous thromboembolism (VTE) [white], VTE [gray], and healthy controls [light gray]. Box and whisker plots displaying medians [mid-line], interquartile range (IQR) [box], 1.5 × IQR [whisker], outliers > 1.5 × IQR [ring], and extreme outliers > 3 × IQR [asterisk].

The pretest probability was 0.38 for all evaluated assays. Box and whisker plots of assay results in patients with VTE, without VTE, and healthy controls are presented in Fig. 2 . All OHP and ETP parameters, as well as fibrinogen, demonstrated prothrombotic profiles in patients with clinically suspected VTE with significant differences compared with healthy controls (Kruskal–Wallis rank-sum test: *p* < 0.005).

Assay differences between patients with and without VTE are presented in Table 2 . Significant differences were found in D-dimer and FM ( *p* < 0.001), ETP T _lag_ ( *p* = 0.031), and for ETP AUC ( *p* = 0.001). ETP AUC was associated with VTE with an OR of 1.04 (95% CI, 1.02–1.07), given a one-unit increase in ETP AUC. OCP was not associated with VTE in univariate analysis, OR 1.03 (95% CI, 0.99–1.08; *p* = 0.17), or after adjusting for fibrinogen levels, OR 0.94 (95% CI, 0.85–1.05; *p* = 0.27). Reference intervals for ETP and OHP 27 31 and the central 95 percentiles of healthy controls are also presented in Table 2 .

Outliers (not excluded, Fig. 2 ) consisted of a patient with large central bilateral PE with high OHP and OCP, and a high outlier in ETP AUC with a recent proximal humerus fracture. ETP C _max_ had a low outlier with hemoglobin 35 and a high outlier with thrombophilia and DVT and ETP T _lag_ had several outliers. ETP T _max_ had two nonpathological outliers, while most other patients had shortened times to peak.

**Fig. 3:**
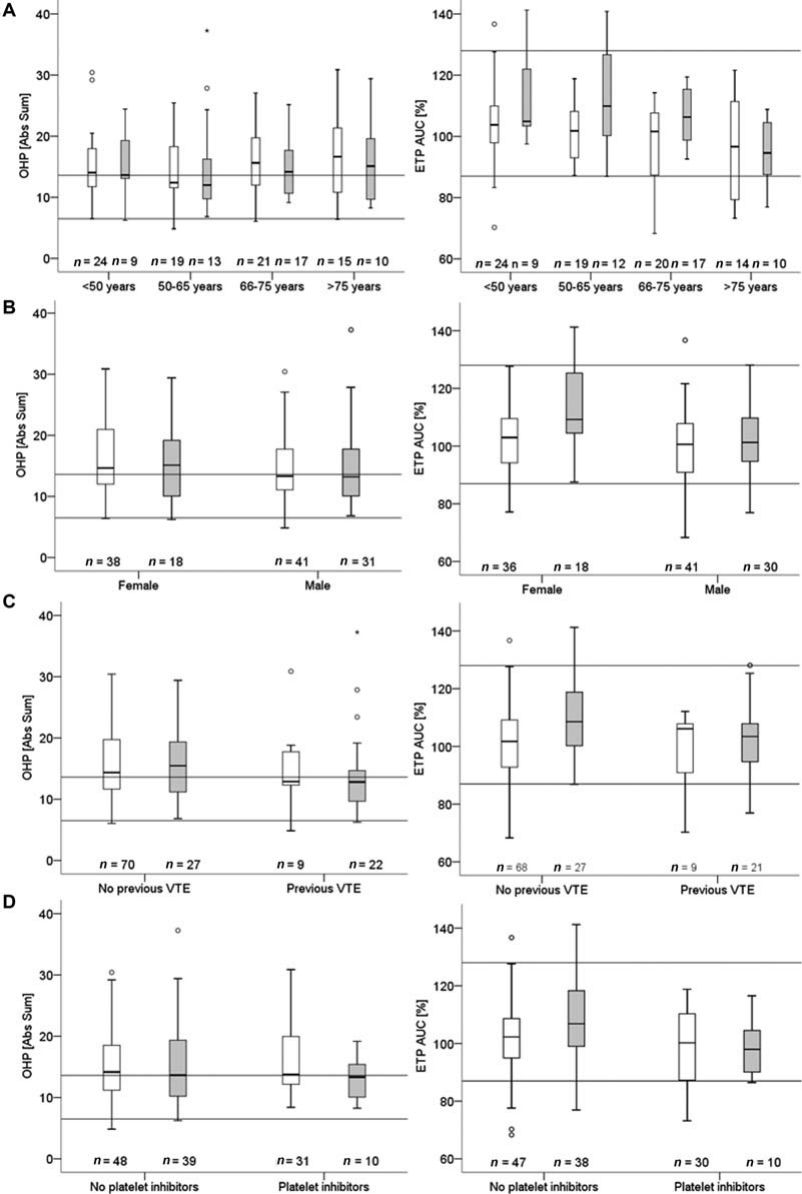
(A) Age, (B) gender, (C) previous venous thromboembolism (VTE), and (D) platelet inhibitors. Box and whisker plots of endogenous thrombin potential area under the curve (ETP AUC) and overall hemostatic potential (OHP) related to age, gender, and previous VTE (samples in analysis = 125). No VTE [white], VTE [gray], medians [mid-line], interquartile range (IQR) [box], 1.5 × IQR [whisker], outliers > 1.5 × IQR [ring], and extreme outliers > 3 × IQR [asterisk].

To investigate potential skewing by third variables, we also assessed the relationship between ETP AUC and OHP levels with age, gender, and previous VTE ( Fig. 3 ). In this analysis, patients with pregnancy, cancer, liver pathology, and recent trauma or surgery were excluded (remainder; *n* = 125). ETP AUC showed a decrease with increasing age, whereas OHP did not correlate with age. ETP AUC was also increased in females, where the difference between patients with and without VTE was more pronounced than in the group as a whole. OHP was less increased in patients with previous VTE than without, regardless of current VTE status. Treatment with platelet inhibitors was significantly more common in controls than in patients but affected neither ETP AUC nor OHP. Due to low numbers, it was not possible to assess the occurrence of any trends in the relationship between ETP AUC or OHP and recent trauma/surgery, cancer, or liver pathology ( *n* = 158) in relation to VTE status ( Supplementary Fig. S1 ).

Finally, considering a full diagnostic model of VTE that included trauma, liver disease, recent pregnancy, thrombophilia, cancer state, gender, and D-dimer, the effect of ETP AUC was not significant (data not presented).

### Assessment of Diagnostic Accuracy of ETP and OHP for VTE

**Fig. 4:**
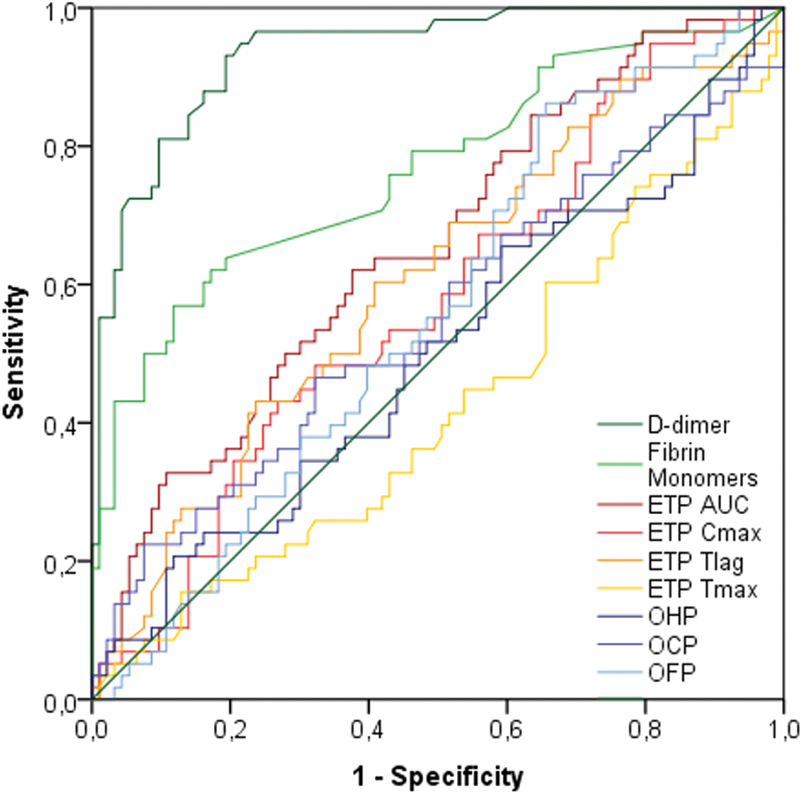
Receiver-operating characteristic (ROC) curves for global hemostatic assays, D-dimer, and fibrin monomers. D-dimer and fibrin monomers are plotted in green, overall hemostatic potential (OHP) parameters in blue, and endogenous thrombin potential (ETP) parameters in red-orange. ETP T _max_ (light orange) is negative because it is the only parameter where a smaller test result indicates a more “positive” test.

For discrimination of radiology-confirmed VTE among patients with suspected VTE, all parameters of OHP and ETP had ROC AUCs ≤ 0.65 ( Table 3 ; Fig. 4 ). The ROC AUC of D-dimer was 0.94 and FM 0.76, while the largest AUC of the global hemostatic parameters was ETP AUC at 0.65. The specificities ranged from 0.00 to 0.20 at the respective cutoffs where the sensitivities of each parameter was 0.97 (in accordance with the Clinical and Laboratory Standards Institute [CLSI] recommendations 38 and equal to the sensitivity of D-dimer in the cohort).

**Table 3:** Diagnostic accuracy of the evaluated assays presented as areas under the ROC curve and as sensitivity and specificity at a cutoff with sensitivity equal to D-dimer and the CLSI requirements for assays to exclude venous thrombosis

Parameter	ROC AUC (95% CI)	Cutoff	Sensitivity	Specificity
OHP [Abs Sum]	0.50 (0.40–0.60)	< 7	0.97	0.04
OCP [Abs Sum]	0.55 (0.45–0.65)	< 12	0.97	0.00
OFP [%]	0.56 (0.47–0.65)	< 17	0.97	0.09
ETP AUC [%]	0.65 (0.56–0.74)	< 85	0.97	0.20
ETP C _max_ [%]	0.57 (0.48–0.67)	< 90	0.97	0.13
ETP T _lag_ [s]	0.60 (0.51–0.70)	< 15	0.97	0.02
ETP T _max_ [s]	0.43 (0.33–0.52)	< 42	0.97	0.01
D-dimer [mg/L FEU]	0.94 (0.90–0.97)	< 0.5	0.97	0.66
Fibrin monomers [mg/L]	0.76 (0.68–0.85)	< 0.9 < 6.0	0.97 0.57	0.20 0.84

Abbreviations: Abs Sum, sum of the absorbances; CI, confidence interval; CLSI, Clinical and Laboratory Standards Institute; ETP, endogenous thrombin potential; FM, fibrin monomers; FEU, fibrin equivalent units; OHP, overall hemostatic potential; ROC AUC, area under the receiver-operating characteristic curve.

Note: For FM, sensitivity and specificity at the current proprietary cutoff (< 6.0 mg/L) is also presented.

Similarly, an exploration of other cutoffs to minimize the distance to the upper left corner of the ROC (the Euclidean distance between the ROC curve and the point where sensitivity and specificity are both 1.0) corresponded to poor tradeoffs of sensitivity and specificity, with sensitivities and specificities in the region of 0.40 to 0.70 ( Table 3 ).

Distal DVT was the main pathology in 32% of the patients with VTE, and low thrombotic load may decrease an evaluated diagnostic sensitivity. When we excluded these patients and restricted analyses to only the cases with proximal DVT and (segmental) PE, the ROC AUC increased by only –0.02 to 0.05 (data not presented). When we excluded the nine patients with thrombophlebitis from the patient controls without VTE, ROC AUC increased by 0.01 for OCP, OFP, and ETP T _lag_ . Differences between patients with and without VTE did not become significant by these restrictions.

### Additive Diagnostic Value after Initial D-Dimer Testing

In the subgroup of patients with positive D-dimer, we could not observe any increase in sensitivity/specificity or ROC AUC of ETP or OHP (data not presented). Given the association of ETP AUC with VTE, we also assessed the potential value of ETP AUC after D-dimer testing. Only two patients with VTE had a negative D-dimer; both had an ETP AUC > 100%. Among patients with a positive D-dimer, 17/37 (46%) patients with ETP AUC < 100% had a VTE; compared with 40/53 (75%) with an ETP AUC ≥ 100%. Although the ETP AUC was associated with VTE in D-dimer-positive patients, it insufficiently discriminated VTE from non-VTE (data not presented).

## Discussion

We performed a cross-sectional single-center diagnostic study to assess the clinical value of two plasma-based GHAs in patients with suspected acute VTE (DVT or PE), compared with D-dimer and FM. We were able to confirm the previously described increase of ETP AUC 8 9 and ETP T _lag_ 8 9 10 11 12 in acute VTE. However, the significant differences were appreciably smaller than for D-dimer and insufficient to discriminate between patients with and without VTE. These results are in line with recent clinical evaluations of other TGA for acute VTE. 10 Our results indicate that neither the ETP nor the OHP assay would be clinically useful additions as biomarkers for the diagnosis of acute VTE in the emergency department.

A biomarker to replace or complement D-dimer would need to exhibit a robust specificity at a cutoff chosen to have at least the same sensitivity as D-dimer. The CLSI recommendations for D-dimer assays for the exclusion of VTE, state that sensitivity at the chosen cutoff must be ≥ 0.97 (≥ 0.90, lower limit 95% CI). 38 Application of this criterion to the GHA demonstrated that none of the ETP and OHP parameters can achieve such sensitivity while maintaining a useful specificity.

Interestingly, patients with suspected VTE showed significantly increased fibrinogen levels compared with the healthy controls, regardless of final VTE status. The OHP and OCP were also increased and OFP was decreased. These results indicate that a large portion of the emergency patients were in a prothrombotic state, which could be explained in part by increased fibrinogen levels. This could be considered a factor that makes the OHP unsuitable for exclusion of VTE in emergency department. The prothrombotic tendency was not observed to the same extent in the ETP assay, which is analyzed in defibrinated samples, although ETP T _max_ and ETP T _lag_ were shortened in both groups and ETP C _max_ was increased. ETP AUC seemed to be least affected, in accordance with studies that suggest it is less influenced by some comorbidities than D-dimer. 7 8 However, ETP AUC was not superior to D-dimer for discrimination of VTE in this real-life cohort with a relatively high comorbidity burden. The use of TGAs in acute settings may indeed be prone to acute phase effects which have not been extensively evaluated yet. One such issue that impacts the accuracy of the TGAs, is the existence of α-2-macroglobulin (α2M)–thrombin complexes in plasma. The Innovance ETP assay attempts to correct for the presence of a fixed amount of α2M–trombin via mathematical calculations, but levels of α2M can vary greatly by age and by conditions such as hepatitis C, pancreatitis, or acute ischemic heart disease, 39 which introduces an interindividual variation in the physiological relevance of TGAs. It is possible that several of the patients in this study had abnormal levels of α2M, which may have influenced the results of the ETP assay.

To evaluate the possible use of Innovance ETP as a second-tier analysis for VTE, we analyzed the diagnostic accuracy in the subgroup of patients with positive D-dimer and observed no increase in sensitivity/specificity or ROC AUC. Haas et al 9 suggested that for patients > 75 years with positive D-dimer and low Wells score, the Innovance ETP T _Lag_ could be used as a second-tier analysis in the emergency department. This was based on a group of 30 patients, where ROC AUC was 0.96 using a cutoff of 23 seconds, sensitivity 1.00, and specificity 0.96. In our cohort, 29 patients were above 75 years with a positive D-dimer. In this group, the ROC AUC for ETP T _Lag_ was 0.49, if the same cutoff of 23 seconds was applied. The sensitivity was only 0.46 paired with a specificity of 0.75 (data not presented). In conclusion, our findings contradict the use of ETP T _Lag_ as a second-tier analysis in patients with unselected clinical probability of VTE.

### Limitations

Only a limited set of matched control samples could be analyzed due to logistical reasons previously explained. However, we minimized the risk of selection bias by choosing pairs at random after matching for age and sex.

Given our study size, it was not possible to stratify patients with PE and proximal or distal DVT. Distal DVT was the main pathology in 32% of the patients with VTE, that is, cases where surveillance is often recommended over anticoagulant treatment, though the vast majority is treated with anticoagulants. 40 However, our results did not change when we excluded patients with distal DVT from the analysis.

The results of the GHA may be affected by the possible presence of thrombophilia in some subjects, although this does not affect the conclusion of the study. It would have been very interesting to acquire information on thrombophilia status in all participating patients, as in the study by Chaireti et al. 11

Optimally, all patients would have been evaluated by diagnostic imaging on the day of sampling. However, since the study was performed within an existing clinical setting, the access to acute appointments for patients with discrete symptoms is somewhat limited and 20 patients were investigated one or more days later. However, we do not expect that this impacted outcomes. In our experience, venous thromboses will not be dissolved in a matter of days, so as to no longer be present in radiological exams. 41 We also performed a 3-month follow-up of medical records, to decrease the risk of false negative diagnoses.

The use of platelet-free plasma is recommended for thrombin generation in hemophilia, 29 because even traces of platelets can lead to overestimation of thrombin generation. This is especially troublesome in patients with low levels of thrombin generation, such as hemophilia patients. Since this study utilized samples collected for routine coagulation assays, these were platelet poor (< 10 × 10 ^9/L^ ), prepared by a stat-protocol with centrifugation at 3,000 × *g* × 10 minutes. However, since all samples were prepared the same way, the study outcome of discrimination between clinical groups was not expected to be affected. In hypocoagulable patients, it is also recommended to use TF in low concentration (≤ 1 M). In this study, we used the Innovance ETP B-setting, which are the proprietary recommended settings for patients with hypercoagulability.

## Conclusion

In this cross-sectional diagnostic study, the OHP and Innovance ETP assays could not discriminate patients with VTE among emergency department outpatients with suspected VTE. The GHAs further indicated that the patients with suspected VTE were in a prothrombotic state, due in part to an increased fibrinogen level. These data suggest that OHP and ETP are sensitive to acute phase effects and comorbidities that are unavoidable in outpatients at the emergency department. In conclusion, the ETP and OHP do not seem to improve the diagnosis of acute VTE.
