# Supplementary material for: Diagnostic Accuracy in Acute Venous Thromboembolism: Comparing D-Dimer, Thrombin Generation, Overall Hemostatic Potential, and Fibrin Monomers
Source: TH Open. 2020 Aug 20;4(3):e178–88. doi: 10.1055/s-0040-1714210 (PMC7440969; doi:10.1055/s-0040-1714210)
Supplement: Supplementary file 1 — Supplementary Material [file 10-1055-s-0040-1714210-s200019.pdf]

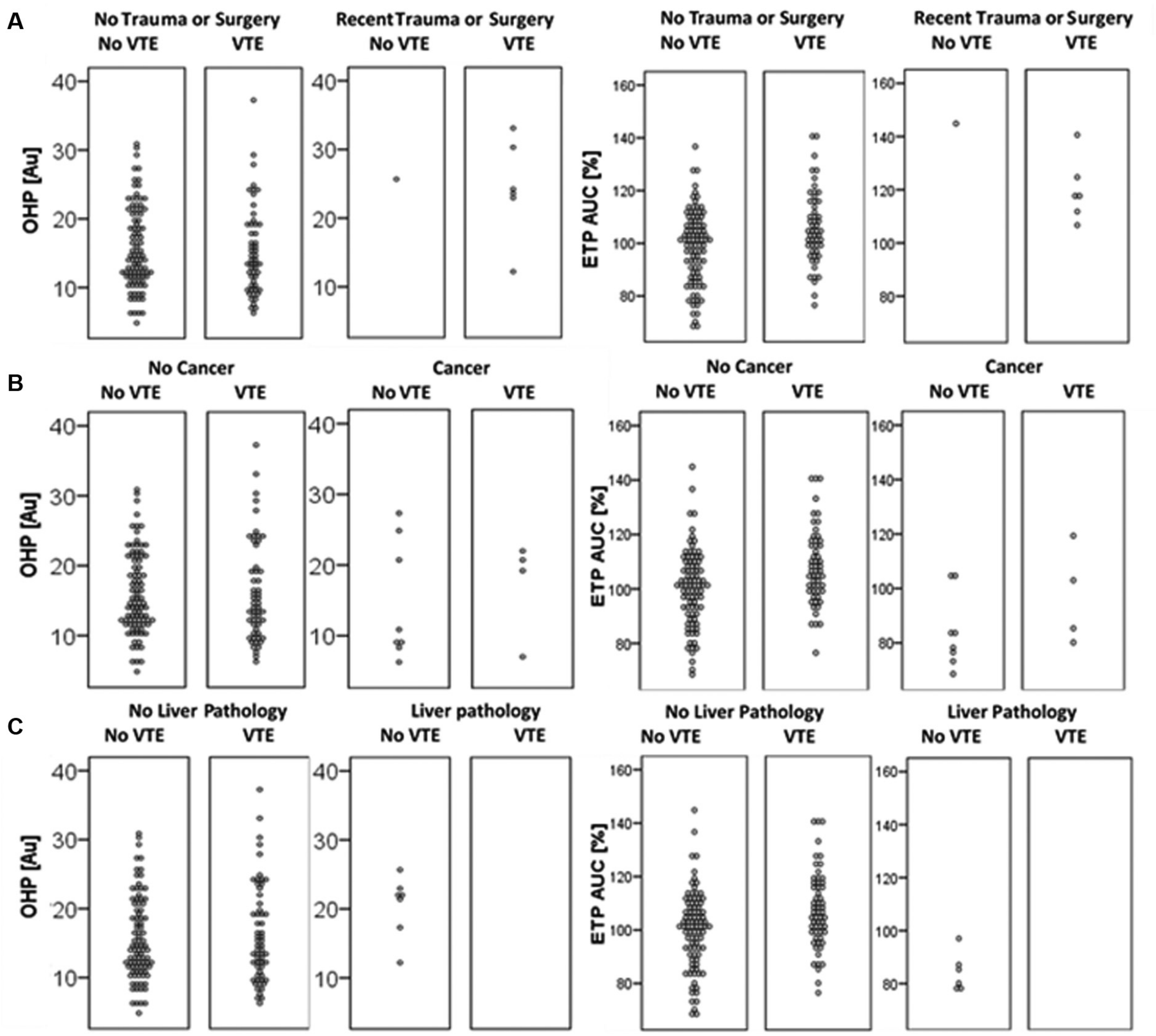

Supplementary Fig. S1 Assay results of overall hemostatic potential (OHP) and endogenous thrombin potential area under the curve (ETP AUC) in clinical subgroups of low frequency traits, too few with venous thromboembolism (VTE) to discern any tendencies. (**A**) Recent trauma/surgery (VTE vs. no VTE, $p = 0.012$). (**B**) Cancer (VTE vs. no VTE, $p = 1.000$). (**C**) Liver pathology (VTE vs. no VTE, $p = 0.045$).
